# Protease activated receptor-1 regulates macrophage-mediated cellular senescence: a risk for idiopathic pulmonary fibrosis

**DOI:** 10.18632/oncotarget.6095

**Published:** 2015-10-12

**Authors:** Cong Lin, Farhad Rezaee, Maaike Waasdorp, Kun Shi, Tom van der Poll, Keren Borensztajn, C. Arnold Spek

**Affiliations:** ^1^ Center for Experimental and Molecular Medicine, Academic Medical Center, Amsterdam, The Netherlands; ^2^ Department of Cell Biology, University Medical Center Groningen, University of Groningen, The Netherlands; ^3^ Inserm UMR1152, Medical School Xavier Bichat, Paris, France; ^4^ Département Hospitalo-universtaire FIRE and LabEx Inflamex, Paris, France

**Keywords:** protease-activated receptor, pulmonary fibrosis, bleomycin, macrophages, cellular senescence, TGF-β, Gerotarget

## Abstract

Idiopathic pulmonary fibrosis (IPF) is a destructive disease in part resulting from premature or mature cellular aging. Protease-activated receptor-1 (PAR-1) recently emerged as a critical component in the context of fibrotic lung diseases. Therefore, we aimed to study the role of macrophages in PAR-1-mediated idiopathic pulmonary fibrosis. The number of macrophages were significantly reduced in lungs of PAR-1 antagonist (P1pal-12) treated animals upon bleomycin instillation. In line with these data, PAR-1 stimulation increased monocyte/macrophage recruitment in response to epithelium injury in *in vitro* trans-well assays. Moreover, macrophages induced fibroblasts migration, differentiation and secretion of collagen, which were inhibited in the presence of TGF-β receptor inhibitors. Interestingly, these profibrotic effects were partially inhibited by treatment with the PAR-1 inhibitor P1pal-12. Using shRNA mediated PAR-1 knock down in fibroblasts, we demonstrate that fibroblast PAR-1 contributes to TGF-β activation and production. Finally, we show that the macrophage-dependent induction of PAR-1 driven TGF-β activation was mediated by FXa. Our data identify novel mechanisms by which PAR-1 stimulation on different cell types can contribute to IPF and identify macrophages as key players in PAR-1 dependent development of this devastating disease. IPF may result from cellular senescence mediated by macrophages in the lung.

## INTRODUCTION

Idiopathic pulmonary fibrosis (IPF) is a devastating aging disease characterized by (myo) fibroblast proliferation and excessive extracellular matrix (ECM) formation leading to destruction of the lung architecture in part as a result of premature or mature cell function reduction [1-5]. The current paradigm proposes that pulmonary fibrosis results from chronic epithelial damage leading to an aberrant wound healing response [6-7]. Although knowledge of the pathogenesis of IPF continues to evolve, therapeutics that effectively improve the clinical outcome of IPF are limited [8]. To date, only pirfenidone and nintedanib delay the destruction of lung function in patients with IPF. However, both drugs have side effects, have no effect on the quality of life, and do not stop nor reverse the disease [9-11]. This indicates that alternative therapeutics are essential for this cell age-related disorder and novel treatment options will only become available due to the comprehensive understanding of the underlying mechanisms like premature/mature cellular senescence in the development of IPF. Also, the discovery of components involved in premature and mature aging may help to prevent premature cellular senescence and restore its regulated function thereby preventing premature lung aging [9].

Protease-activated receptor (PAR)-1 is a cell surface seven-transmembrane G protein-coupled receptor that is activated by proteolytic cleavage, inducing transmembrane signaling to intracellular G proteins leading to a broad range of pathophysiological pathways [12]. Importantly, PAR-1 recently emerged as a critical component in the context of fibrotic lung disease. Indeed, PAR-1 expression is increased within fibroproliferative and inflammatory foci in IPF [13]. PAR-1 activation also stimulates fibroblast differentiation and ECM production [14, 15]. Moreover, PAR-1 seems to synergize with PAR-3 to mediate epithelial-mesenchymal transition of alveolar epithelial cells [16]. In line with these *in vitro* data, PAR-1 deficiency in mice limits bleomycin-induced pulmonary fibrosis whereas pharmacological inhibition of PAR-1 also limits bleomycin-induced pulmonary fibrosis [13, 14].

Interestingly, PAR-1 overexpression is found in alveolar macrophages from patients with chronic airway disease and PAR-1 expression in IPF patients is associated with macrophages [13, 17]. This may be particularly important as macrophages are known to be key regulators in the progression of pulmonary fibrosis [18-20]. In this context, macrophage influx is an early event following lung injury and macrophages secrete large amounts of profibrotic cytokines like transforming growth factor-β (TGF-β) [21]. TGF-β on its turn induces fibroblast proliferation and differentiation into myofibroblasts leading to ECM deposition thereby promoting fibrosis [20].

In the present study, we aimed to address the potential importance of macrophages in PAR-1-dependent pulmonary fibrosis. We show that PAR-1 modifies macrophage recruitment to the lung during pulmonary fibrosis, and we identify a potential mechanism by which PAR-1 mediates macrophage induced profibrotic responses.

## RESULTS

### PAR-1 regulates monocyte/macrophage recruitment during pulmonary fibrosis

**Figure 1:**
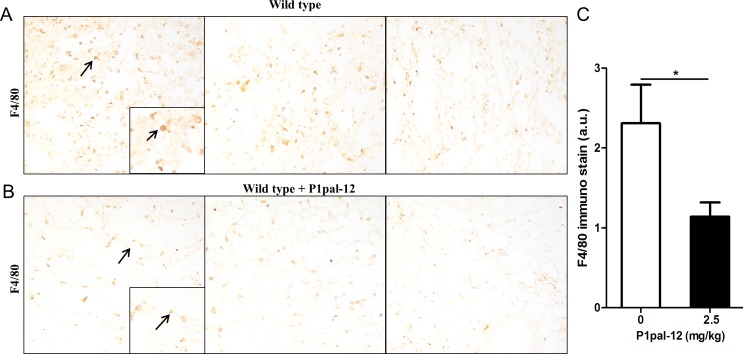
PAR-1 inhibition reduces macrophage numbers in the lung of bleomycin treated mice Representative macrophage marker F4/80 stained sections obtained 14 days after bleomycin instillation in wild type mice **A.** and wild type mice treated with the PAR-1 inhibitor P1pal-12 (2.5 mg/kg) **B.** The arrows indicate an example of F4/80 positive macrophages. **C.** Quantification of macrophage numbers in fibrotic mice treated or not with P1pal-12 (mean±SEM, *n* = 8 mice per group). *P < 0.05.

As macrophage recruitment in response to chemoattractant production by injured epithelial cells is a key process in fibrosis, we set out to determine whether PAR-1 would modify macrophage recruitment into fibrotic lungs. As shown in Figure 1A, macrophages were omnipresent in lungs of wild type mice subjected to bleomycin-induced pulmonary fibrosis as evident from large amounts of F4/80 (ADGRE1) positive cells. Interestingly, macrophage numbers were reduced by approximately 50% in fibrotic mice treated with the PAR-1 inhibitor P1pal-12 (Figure 1B, 1C).

**Figure 2:**
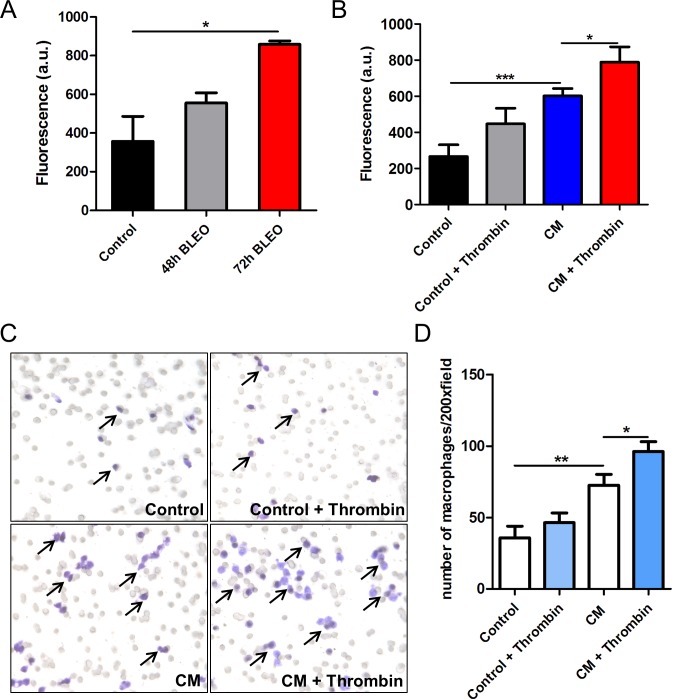
PAR-1 regulates macrophages migration in trans-well assays **A.** Migration of RAW264.7 cells towards epithelial cell conditioned medium (collected after exposure to 10 μg/ml bleomycin for 48 or 72 hours) for 10 hours. RAW264.7 cell migration towards plain medium was used as control. **B.** Migration of RAW264.7 cells towards control or MLE-15 conditioned medium (10 μg/ml bleomycin for 72 hours) for 10 hours in the absence or presence of thrombin (1 U/ml). Shown is the mean ± SEM, *n* = 3. **C.** Representative pictures of RAW264.7 cells migrated through the trans-well toward plain control or MLE-15 epithelial cells conditioned medium (CM) stimulated with or without thrombin (1 U/ml). **D.** Quantification of the data presented in **C.** (mean ± SEM of an experiment performed three times, *P < 0.05 and **P < 0.01).

To assess whether the reduced macrophage numbers in P1pal-12 treated mice are due to a direct effect of PAR-1 on the migration of macrophages towards injured epithelium, the migration of RAW264.7 macrophages was measured *in vitro*. To mimic the *in vivo* setting, lung epithelial cells were exposed to bleomycin (10 μg/ml) for 48 or 72 hours after which the medium was used as chemoattractant for RAW264.7 cells. As shown in Figure 2A, medium of bleomycin-exposed MLE-15 cells indeed served as a chemoattractant for RAW264.7 cells. Stimulation of RAW264.7 cells with the PAR-1 agonist thrombin did not have any effect on migration towards control medium, but potentiated migration towards bleomycin-treated MLE-15 conditioned medium (Figure 2B-2D). These results thus indicate that macrophage recruitment into injured lungs seems (at least in part) PAR-1 dependent.

### Macrophages induce fibrotic responses in fibroblasts via TGF-β in a PAR-1 dependent manner

**Figure 3:**
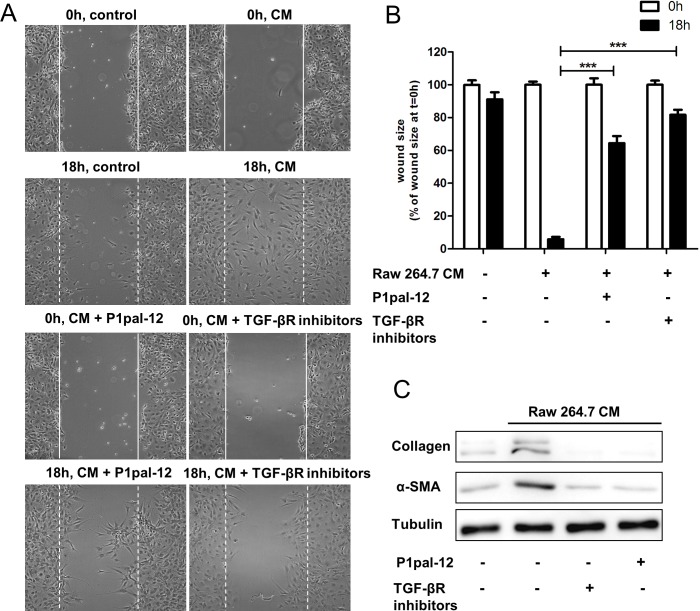
Macrophages-induced fibrotic responses of fibroblasts are PAR-1 dependent **A.** Wound size of NIH3T3 fibroblast monolayers after treatment with PBS (control) or RAW264.7 conditioned medium (CM) for 18 hours in the presence or absence of TGFBR inhibitors (10 μM SB-431542 and 10 μM LY-2157299) or P1pal-12 (10 μM). Shown are photographs of representative microscopic fields. **B.** Quantification of the results depicted in **A.** as described in the Materials and Methods section. Data are expressed as mean±SEM (*n* = 6 independent experiments). ***P < 0.001. **C.** Western blot analysis of α-SMA and collagen expression in NIH3T3 cells stimulated with RAW264.7 CM in the absence or presence of P1pal-12 (10 μM) or TGFBR inhibitors (10 μM of SB-431542 and 10 μM of LY-2157299). Tubulin served as loading control. Gels have been run under the same experimental conditions and uncropped blots are shown in Supplementary Figure.

To assess whether the decreased number of macrophages in lungs of P1pal-12 treated mice correlate with the observed reduction in fibrosis, we subsequently analyzed macrophage-induced profibrotic responses in fibroblasts. RAW264.7 conditioned medium induced fibroblast migration as demonstrated by efficient wound closure which is not observed in the control medium (Figure 3A-3B). In line, RAW264.7 conditioned medium also induced fibroblast differentiation and ECM production as evident from increased alpha-smooth muscle actin (α-SMA; ACTA2) and collagen I expression levels (Figure 3C). To determine whether the macrophage-induced profibrotic responses of fibroblasts rely upon PAR-1 activation on fibroblasts, we next pre-incubated fibroblasts with P1pal-12 before assessing the macrophage-dependent fibrotic responses. As shown in Figure 3, P1pal-12 treatment significantly inhibited macrophage-induced wound closure, fibroblast differentiation and collagen deposition suggesting macrophages potentiate fibroblast-driven fibrosis in a PAR-1 dependent manner.

Next, we determined whether TGF-β plays a dominant role in the pro-fibrotic effects of RAW264.7 conditioned medium. We assessed macrophage-dependent fibroblast migration in the presence of TGF-β receptor inhibitors. As shown in Figure 3A-3B, inhibition of TGF-β signaling inhibited RAW264.7 conditioned medium-induced wound closure. Consistently, TGF-β receptor (TGFBR) inhibition also prevented macrophage-induced fibroblast differentiation (i.e. α-SMA expression) and ECM deposition (i.e. collagen production) (Figure 3).

**Figure 4:**
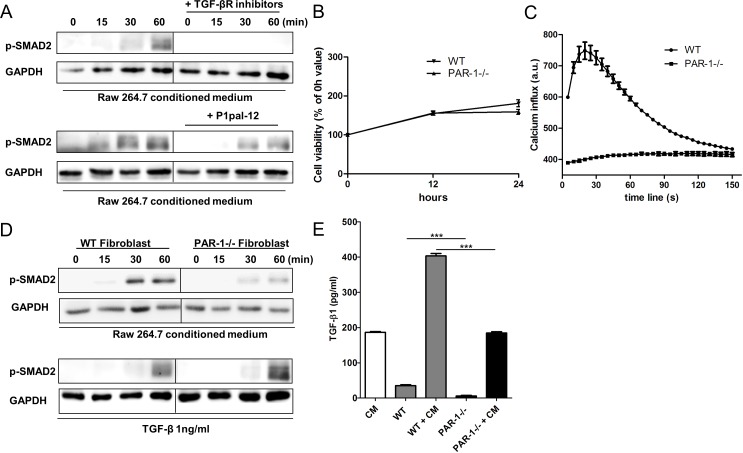
PAR-1 mediates TGF-β activation and production **A.** Representative Western blot analysis of SMAD2 phosphorylation in NIH3T3 cells stimulated for 0, 15, 30 and 60 minutes with RAW264.7 CM in the absence or presence of TGFBR inhibitors (10 μM SB-431542 and 10 μM LY-2157299) or P1pal-12 (10 μM). GAPDH served as loading control. **B.** Cell viability of NIH3T3s lentivirally transduced with a control shRNA construct (WT fibroblasts, down-pointing triangle) or a PAR-1 shRNA construct (PAR-1−/− fibroblasts, up-pointing triangle) as evaluated by MTT assays after 12 or 24 hours of incubation (Mean+/−SEM of an experiment performed two times in octoplo). **C.** Intracellular Ca^2+^ fluxes in WT fibroblasts (circle) and PAR-1−/− fibroblasts (square) after stimulation with thrombin (1 U/ml). Ca^2+^ fluxes are expressed as arbitrary units of fluorescent intensity after background correction. Shown is a representative experiment of three independent experiments. **D.** Western blot analysis of SMAD2 phosphorylation in WT fibroblasts or PAR-1−/− fibroblasts stimulated for 0, 15, 30 and 60 minutes with RAW264.7 CM or recombinant TGF-β (1 ng/ml). GAPDH served as loading control. **E.** Total TGF-β production of RAW264.7 cells and WT or PAR-1−/− fibroblasts stimulated without or with RAW264.7 conditioned medium (CM) after 24 hours (mean ± SEM, *n* = 6; ***P < 0.001).

To confirm these findings, we assessed macrophage-induced SMAD2 phosphorylation, a direct downstream target of TGFBR activation in fibroblasts. As shown in Figure 4A, RAW264.7 conditioned medium clearly caused a time-dependent increase in SMAD2 phosphorylation in fibroblasts. Notably, SMAD2 phosphorylation was completely blocked by TGF-β receptor inhibitors. SMAD2 phosphorylation was also partly blocked by the PAR-1 inhibitor P1pal-12. This suggests that TGF-β signaling on fibroblasts is in part mediated by PAR-1.

To evaluate the P1pal-12 data and to elucidate the mechanism by which PAR-1 influences TGF-β signaling, we generated a stable PAR-1 knockdown fibroblast cell line. As shown in Figure 4B, fibroblasts lentivirally transduced with PAR-1 shRNA (indicated as PAR-1−/− cells) proliferated to a similar extent as fibroblasts transduced with control shRNA (indicated as WT cells). However, the PAR-1−/− cells did not respond to thrombin stimulation in calcium assays as opposed to wildtype (WT) cells confirming efficient knock-down (Figure 4C). As expected, WT fibroblasts showed increased SMAD2 phosphorylation after stimulation with RAW264.7 conditioned medium (Figure 4D). In line with p1pal-12 treatment, PAR-1 knock-down significantly inhibited macrophage-induced SMAD2 phosphorylation (Figure 4D). Notably, PAR-1−/− fibroblasts still responded to direct TGF-β stimulation and showed similar SMAD2 levels as WT fibroblasts (Figure 4D). Our data thus show that PAR-1 on fibroblasts modifies TGF-β signaling most likely by regulating the activation of latent TGF-β.

Finally, we assessed whether fibroblast PAR-1 would also modify TGF-β production induced by macrophage-conditioned medium. As shown in Figure 4E, baseline latent TGF-β levels were already reduced in PAR-1−/− fibroblasts as compared to WT fibroblasts. Moreover, RAW264.7 conditioned medium induced TGF-β expression by WT cells but not by PAR-1−/− cells (TGF-β level in PAR1−/− cells stimulated with RAW264.7 conditioned is similar to the levels in conditioned medium alone). It seems that PAR-1 expression on fibroblasts also potentiates TGF-β production.

### Factor Xa as PAR-1 agonist secreted by RAW264.7 cells

**Figure 5:**
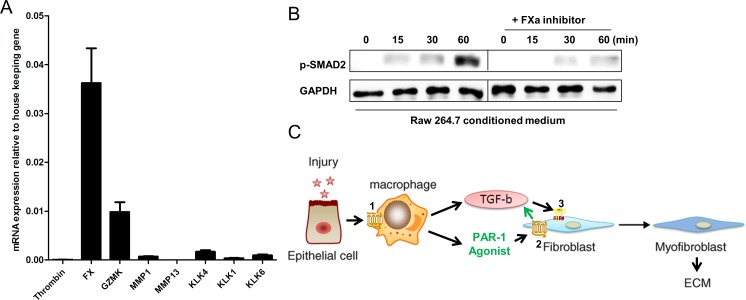
PAR-1-induced TGF-β activation on fibroblasts is mediated by FX **A.** Thrombin (FII), FX, Granzyme K (GZMK), MMP1, MMP13, KLK1, KLK4 and KLK6 mRNA levels in RAW264.7 cells as assessed by real-time reverse transcriptase PCR. Data are expressed relative to two housekeeping genes, GAPDH and TBP. Shown is the mean ± SEM, of an experiment performed three times. **B.** Western blot analysis of SMAD2 phosphorylation in NIH3T3 cells stimulated for 0, 15, 30 and 60 minutes with RAW264.7 CM in the absence or presence of the FXa inhibitor antistasin (40 μM). GAPDH served as loading control. **C.** Proposed mechanism by which macrophages promote lung fibrosis in a PAR-1 dependent manner. During lung injury, epithelial cells release mediators that potentiate PAR-1 dependent macrophage migration towards the injured site (1). The recruited macrophages subsequently secrete TGF-β and FX. The PAR-1 agonist (FX) than activates PAR-1 on fibroblasts (2) leading to TGF-β production and activation. Finally, TGF-β induces TGFBR signaling (3) on fibroblast thereby inducing their migration, differentiation and ECM deposition.

The data obtained suggest a crosstalk between fibroblasts and macrophages, where macrophages are a source of one or several PAR-1 agonist(s) that subsequently stimulate fibroblasts leading to TGF-β production and activation. Hence, to identify the potential PAR-1 agonist, we first analyzed mRNA levels of known PAR-1 agonists in RAW264.7 cells. Interestingly, Granzyme K and particularly factor X (FX) are significantly expressed by RAW264.7 macrophages, whereas matrix metallopeptidase (MMP)-1, kallikrein (KLK)-1, -4 and -6 were expressed at relatively low levels. To prove or refute that FX is the PAR-1 agonist secreted by RAW264.7 cells, we next determined RAW264.7 cell-induced TGF-β signaling by assessing SMAD2 phosphorylation in fibroblasts in the absence or presence of antistasin, a direct FXa inhibitor. As shown in Figure 5B, RAW264.7 conditioned medium-induced SMAD2 phosphorylation was almost completely blocked by antistasin pinpointing FXa as endogenous PAR-1 ligand secreted by Raw264.7 cells.

## DISCUSSION

Cell function reduction is considered as a hallmark of cell aging, which is classified into either premature or mature cell aging. During premature and mature cellular senescence, IPF may occur. Epidemiological and biological studies suggest that IPF is an aging-related disease [2, 22-24]. There is compelling evidence that PAR-1 plays an important role in mediating pro-fibrotic effects and pharmacological inhibition of PAR-1 limits bleomycin-induced pulmonary fibrosis [13-15]. The underlying mechanism by which PAR-1 modulates pulmonary fibrosis is however not yet fully understood. In the current manuscript, we identify macrophages as key players in PAR-1 driven pulmonary fibrosis. We show that PAR-1 on macrophage potentiates recruitment of macrophages towards injured lung epithelial cells. Recruited macrophages subsequently secrete PAR-1 agonists that stimulate fibroblasts to produce and activate latent TGF-β leading to fibroblast migration, differentiation into myofibroblasts and ECM deposition (Figure 5C).

A key finding of our study is the fact that macrophage numbers are significantly reduced in fibrotic lungs as a consequence of pharmacological PAR-1 inhibition. To evaluate whether PAR-1 directly modifies macrophage migration, we simulated the *in vivo* condition by analyzing macrophage migration towards conditioned medium obtained from bleomycin-treated lung epithelial cells in trans-well assays. Interestingly, PAR-1 activation by thrombin did not affect macrophage migration towards control medium, but did potentiate directed migration towards conditioned medium of injured epithelial cells. This suggests that PAR-1 specifically modifies chemotaxis of macrophages in the setting of pulmonary fibrosis. The underlying mechanism by which PAR-1 modifies chemotaxis remains unclear although a recent study elegantly shows that PAR-1 activation by thrombin on human monocytes (THP-1 cells) leads to cytoskeletal remodeling and migration [25].

Macrophages recruited to injured lung tissue contribute to the development of fibrosis by secreting the profibrotic cytokine TGF-β that, once activated, targets fibroblasts [18-21]. Our study shows that macrophage TGF-β induces fibroblast migration, differentiation and ECM deposition. Of note, macrophages-induced pro-fibrotic responses were inhibited by a TGFBR blocking cocktail, resulting in inactivation of both TGF-β receptor I (TGFBRI) and II (TGFBRII). Interestingly, all macrophage conditioned medium-induced pro-fibrotic responses were also partially inhibited by the specific PAR-1 inhibitor P1pal-12 which may suggest that PAR-1 directly regulates TGF-β receptor signaling. However, TGF-β is still efficiently triggering TGF-β receptor dependent Smad2 phosphorylation in PAR-1 knock down cells. This suggests that PAR-1 is not required when TGF-β is activated. Most likely, PAR-1 contributes to TGF-β activation on fibroblasts and indeed thrombin-dependent PAR-1 cleavage leads to TGF-β activation on respiratory epithelial cells [26]. It seems that PAR-1 activation induces actin polymerization with subsequent conformational changes in the integrin/latent TGF-β complex that allows the interaction between active TGF-β and its adjacent receptors [27, 28]. Additionally, blocking integrin signaling inhibited TGF-β activation during acute lung injury, confirming the importance of this PAR-1-dependent TGF-β activation pathway *in vivo* [26].

Our data show that macrophage conditioned medium contains a PAR-1 ligand leading to PAR-1 dependent activation of latent TGF-β. We identified FXa as the most likely endogenous agonist secreted by macrophages (consistent with the previous study [29]) based on our data showing that a specific FXa inhibitor blocked macrophage conditioned medium-induced TGF-β signaling. Notably, FXa plays an important role in the pathogenesis of pulmonary fibrosis via enhancement of TGF-β activation in a PAR-1 and integrin-dependent manner. In this regard, FXa inhibition limited bleomycin-induced pulmonary fibrosis [30]. In addition to FXa, other PAR-1 agonists may also be able to contribute to TGF-β activation. Macrophages also express relatively high mRNA levels of Granzyme K, known to induce pro-inflammatory cytokine secretion and lung fibroblast proliferation through PAR-1 [31]. Although all other potential PAR-1 agonists only showed low expression levels in RAW264.7 cells in our experiments, these proteases may obviously not be ruled out as key players in PAR-1 driven pulmonary fibrosis.

Intriguingly, PAR-1 appeared to be important for TGF-β secretion by fibroblasts. Although fibroblasts produce relatively low levels of latent TGF-β as compared to macrophages, PAR-1 knock down fibroblasts produced significantly less latent TGF-β than PAR-1 expressing fibroblasts in unstimulated conditions. This suggests that PAR-1 may actually be essential for fibroblasts to secrete latent TGF-β. Moreover, fibroblast stimulation by macrophages conditioned medium (containing PAR-1 agonists as discussed above) induced secretion of latent TGF-β in wild type but not PAR-1 knock down fibroblasts. Our data confirm previous findings that activation of PARs may indeed lead to cytokine release and TGF-β production [32-33]. The role of PAR-1 in TGF-β expression by fibroblasts and the potential effect of fibroblast TGF-β in pulmonary fibrosis remains to be elucidated.

In conclusion, PAR-1 stimulation on different cell types may contribute to pulmonary fibrosis and we propose a PAR-1-mediated pathogenesis of pulmonary fibrosis (Figure 5C). We show that macrophages play a crucial role in PAR-1 dependent pulmonary fibrosis and we suggest that macrophages secrete FXa that targets fibroblasts to enhance TGF-β driven fibrotic effects.

## MATERIALS AND METHODS

### Reagents

Thrombin and bleomycin were from Sigma (St-Louis, MO), recombinant TGF-β was from Tebu-bio (Heerhugowaard, Netherlands), the specific FXa inhibitor antistasin-related peptide was purchased from Bachem (Bubendorf, Switzerland), TGFBRI and TGFBRII were inhibited by a combination of inhibitors SB-431542 and LY-2157299, which were from Axon medchem (Groningen, Netherlands) whereas PAR-1 inhibitor P1pal-12 (palmitate-RCLSSSAVANRS-NH2) was from GL Biochem Ltd (Shanghai, China).

### Cell lines and conditioned medium preparation

Murine NIH3T3 fibroblasts and RAW264.7 macrophages were cultured in DMEM and IMDM, respectively, supplemented with 10% fetal calf serum (FCS). Murine lung epithelial cells (MLE-15) were cultured in HITES medium (RPMI supplemented with 5 μg/ml insulin, 10 μg/ml transferrin, 10 nM estradiol and 10 nM hydrocortisone). Cells were grown at 37°C in an atmosphere of 5% CO_2_. Unless indicated otherwise, cells were washed twice with PBS and serum-starved for 4 hours before stimulation. For conditioned medium preparations, cells were seeded and grown overnight under normal growth conditions to reach subconfluency. Next, the cells were washed with PBS and incubated for 24 hours in FCS free medium with or without the indicated agonists. Finally, the collected media were centrifuged, put through a 0.2 μm filter and stored at −20°C.

### Animal model of pulmonary fibrosis

Ten-week-old C57Bl/6 mice, purchased from Charles River (Someren, Netherlands), were subjected to bleomycin-induced pulmonary fibrosis as described in our previous study [14]. In detail, bleomycin (Sigma, St-Louis) was administered intranasally (1 mg/kg) under anesthesia. Animals were instilled with 2.5 mg/kg P1pal-12 30 minutes before bleomycin administration and subsequently once daily until the end of the experiment. 6% DMSO in saline was administered as solvent control. Mice were sacrificed 14 days after bleomycin instillation, after which one lung was taken for histology and one was homogenized. All procedures were performed in accordance with the Institutional Standards for Humane Care and Use of Laboratory Animals. Experiments were approved by the Animal Care and Use Committee of the Academic Medical Center Amsterdam.

### Lentiviral knockdown of PAR-1

PAR-1 knock down cells were established as described before [34]. Briefly, PAR-1 (clone TRCN0000026806) and control (clone SHC004) shRNA in the pLKO.1-puro backbone were purchased from Sigma-Aldrich (St. Louis, MO; MISSION shRNA library). Lentiviral production and subsequent cell transduction was performed using standard protocols [32]. shRNA transduced NIH3T3 cells were selected in the presence of 5 μg/ml puromycin for 72 h.

### Cell viability assays

Cells were seeded in 96-well plates at a concentration of 5000 cells/well, after which cell viability was determined using a 3-(4,5-dimethylthiazol-2-yl)-2,5-diphenyltetrazolium (MTT) assay at 12 and 24 hours according to routine procedures.

### Calcium assay

Calcium signaling responses were analyzed using the Fluo-4 Direct™ Calcium Assay Kit (Invitrogen, Carlsbad, CA) following the manufacturer's instructions. Cells were challenged with thrombin. Ca^2+^ flux was monitored for the indicated time points on a Bio-Tek HT Multi-Detection Microplate Reader (Winooski, United States).

### Wound scratch assay

Scratch assays were performed essentially as described before [14]. Briefly, fibroblasts were seeded in six-well plates in DMEM supplemented with 10% FCS. After the cells formed a confluent monolayer, a scratch was created in the center of the monolayer by a sterile p200 pipette tip. Next, medium was removed and cells were washed with serum-free medium to remove floating debris. The cells were subsequently incubated for 18 hours with serum-free medium (negative control), RAW264.7 conditioned medium or RAW264.7 conditioned medium containing 10 μM of each TGF-β receptor inhibitor or 10 μM P1pal-12. When indicated, cells were pre-incubated with 10 μM P1pal-12 for 30 minutes before scratching. The ability of cells to close the wound was assessed by comparing the 0- and 18-hour phase-contrast micrographs of 6 marked points along the wounded area. The percentage of non-recovered wound area was calculated by dividing the non-recovered area after 18 hours by the initial area at 0 hour as previously described.

### Western blot

Fibroblasts were seeded in 12-well plates in DMEM supplemented with 10% FCS. Next, medium was removed and cells were washed with serum-free medium. After serum starvation for 4 hours, the cells were incubated with serum-free medium (negative control) or RAW264.7 conditioned medium with or without 10 μM of each TGF-β receptor inhibitor or 10 μM P1pal-12. When indicated, cells were pre-incubated with 10 μM P1pal-12 for 30 minutes. Twenty four hours later, cells were lysed in Laemmli lysis buffer and Western blots were performed as described before [14]. In brief, protein samples were separated by 10% SDS gel electrophoresis and transferred to a PVDF membrane (Millipore, Billerica, MA). Membranes were blocked for 1 hour in 4% milk in TBST and incubated overnight with monoclonal antibodies against a-SMA (1:1000, Santa Cruz, CA), GAPDH (1:1000, Santa Cruz, CA), collagen (1:800, SouthernBiotech, AL) or p-SMAD2 (1:1000, Cell Signaling Technology, Boston, MA) at 4°C. All secondary antibodies were horseradish peroxidase (HRP)-conjugated from DakoCytomation (Glostrup, Denmark) and diluted according to the manufacturer's instructions. Blots were imaged using Lumilight plus ECL substrate from Roche (Almere, The Netherlands) on an ImageQuant LAS 4000 biomolecular imager from GE Healthcare (Buckinghamshire, U.K).

### Trans-well migration assays

Serum starved Raw264.7 cells (1×10^5^ CellTrace CFSE labeled for real-time analysis or 2×10^4^ unlabeled for analysis by microscopy) were transferred to 8 μm pore-size Cell Culture inserts coated with 0.1% (w/v) collagen. The cells were incubated in serum-free medium with or without thrombin, and the inserts were incubated at 37°C for 10 hours in serum-free medium or MLE-15 conditioned medium as chemoattractant. For real time analysis, fluorescence values representing the number of cells on the bottom side of the insert were read on a BioTek plate reader at 485/528 nm (BioTek^®^, Bad Friedrichshall, Germany). For microscopic analysis, cells on the upper side of the transwell membrane were removed with a cotton swab after which the inserts were fixed and stained in a crystal violet solution as described before [34]. The membranes were subsequently mounted on a glass slide, and migrated cells were counted by light microscopy. Cells were counted in five different fields using a 200× magnification.

### Immunohistological analysis

Four-μm sections were deparaffinized and rehydrated. Endogenous peroxidase activity was quenched with 0.3% H_2_O_2_ in methanol. F4/80 staining was performed using an anti-F4/80 antibody (1:500, 24 hour at 4°C, AbD Serotec, Kidlington, UK). A horseradish peroxidase-conjugated polymer detection system (ImmunoLogic, Duiven, the Netherlands) was applied for visualization, using an appropriate secondary antibody and diaminobenzidine (DAB) staining. Slides were photographed with a microscope equipped with a digital camera (Leica CTR500). Pictures of F4/80 stainings were taken to cover the entirety of all sections. Color intensity of stained areas was analyzed semi-quantitatively with ImageJ and expressed as percentage of the surface area essentially as described before [35].

### TGF-β ELISA

TGF-β1 was measured with the Mouse TGF-β1 DuoSet kit (R&D systems, UK) as suggested by the manufacturer.

### Quantitative real-time PCR

Total RNA was isolated from cells with TriPure (Roche, Almere, Netherlands) following the manufacturer's recommendations. q-PCR was performed with SYBR Green PCR master Kit (Roche) using the following primers: Thrombin (FII): forward 5′-GGCAACCTAGAGCGTGAGT-3′ and reverse 5′-TAGCACAGCGACCTTCCAGA-3′; FX: forward 5′-GACCAAATATAAAGACGGCGAC-3′ and reverse 5′-TCCGAACAAAGAGCTCACAGT-3′; Granzyme K: forward 5′-TGAGCCCATGAAGCAGACAT-3′ and reverse 5′-TGGCATTTGGTCCCATCTCT-3′; Matrix metalloproteinases(MMP)-1: forward 5′-TTACGGCTCATGAACTGGGT-3′ and reverse 5′-GTTGGCTGGATGGGATTTGG-3′; MMP-13: forward 5′-AACATCCATCCCGTGACCTT-3′ and reverse 5′-TTCTCAAAGTGAACCGCAGC-3′; KLK-1: forward 5′-CCCACAACCTGAGGATGACT-3′ and reverse 5′-GCTTGAGGTTCACACACTGG-3′; KLK-4: forward 5′-ATCTCTCAGTGGCGTCAGAG-3′ and reverse 5′-CTGCCCACACTTTCCTTGTC-3′; KLK-6: forward 5′-TGGTGTCATTCCCCTCCAAC-3′ and reverse 5′-CCATGAACCACCTTCTCCTGT-3′. GAPDH: forward 5′-CTCATGACCACAGTCCATGC-3′ and reverse 5′-CACATTGGGGGTAGGAACAC-3′; TBP: forward 5′-GGAGAATCATGGACCAGAACA-3′ and reverse 5′-GATGGGAATTCCAGGAGTCA-3′.

The qPCR data were normalized to the average of the housekeeping genes GAPDH and TBP.

### Statistics

Statistical analyses were conducted using GraphPad Prism version 5.00, (GraphPad software, San Diego, CA, USA). Data were expressed as means ± SEM. Comparisons between two conditions were analyzed using two tailed unpaired *t*-tests when the data where normally distributed, otherwise Mann-Whitney analysis was performed. *P* values of less than 0.05 were considered as significant.

## References

[1] King TE, Pardo A, Selman M (2011). Idiopathic pulmonary fibrosis. Lancet.

[2] Collard HR (2010). The age of idiopathic pulmonary fibrosis. Am J Respir Crit Care Med.

[3] Blagosklonny MV (2012). Answering the ultimate question “what is the proximal cause of aging?”. Aging (Albany NY).

[4] Blagosklonny MV (2012). Common drugs and treatments for cancer and age-related diseases: revitalizing answersto NCI's provocative questions. Oncotarget.

[5] Rezaee F (2015). Systems Biology and Age-Induced Diseases. J Diabetes Metab.

[6] Wynn TA (2011). Integrating mechanisms of pulmonary fibrosis. J Exp Med.

[7] Strieter RM, Mehrad B (2009). New mechanisms of pulmonary fibrosis. Chest.

[8] Wuyts WA, Antoniou KM, Borensztajn K, Costabel U, Cottin V, Crestani B, Grutters JC, Maher TM, Poletti V, Richeldi L, Vancheri C, Wells AU (2014). Combination therapy: the future of management for idiopathic pulmonary fibrosis?. Lancet Respir Med.

[9] Raghu G, Selman M (2015). Nintedanib and pirfenidone. New antifibrotic treatments indicated for idiopathic pulmonary fibrosis offer hopes and raises questions. Am J Respir Crit Care Med.

[10] Richeldi L, du Bois RM, Raghu G, Azuma A, Brown KK, Costabel U, Cottin V, Flaherty KR, Hansell DM, Inoue Y, Kim DS, Kolb M, Nicholson AG (2014). Efficacy and safety ofnintedanib in idiopathic pulmonary fibrosis. N Engl J Med.

[11] King TE, Bradford WZ, Castro-Bernardini S, Fagan EA, Glaspole I, Glassberg MK, Gorina E, Hopkins PM, Kardatzke D, Lancaster L, Lederer DJ, Nathan SD, Pereira CA (2014). A phase 3 trial of pirfenidone in patients with idiopathic pulmonary fibrosis. N Engl J Med.

[12] Coughlin SR (2000). Thrombin signalling and protease-activated receptors. Nature.

[13] Howell DC, Johns RH, Lasky JA, Shan B, Scotton CJ, Laurent GJ, Chambers RC (2005). Absence of proteinase-activated receptor-1 signaling affords protection from bleomycin-induced lung inflammation and fibrosis. Am J Pathol.

[14] Lin C, Duitman J, Daalhuisen J, Ten Brink M, von der Thüsen J, van der Poll T, Borensztajn K, Spek CA (2014). Targeting protease activated receptor-1 with P1pal-12 limits bleomycin-induced pulmonary fibrosis. Thorax.

[15] Bogatkevich GS, Tourkina E, Silver RM, Ludwicka-Bradley A (2001). Thrombin differentiates normal lung fibroblasts to a myofibroblast phenotype via the proteolytically activatedreceptor-1 and a protein kinaseC-dependent pathway. J Biol Chem.

[16] Wygrecka M, Didiasova M, Berscheid S, Piskulak K, Taborski B, Zakrzewicz D, Kwapiszewska G, Preissner KT, Markart P (2013). Protease-activated receptors (PAR)-1 and -3 drive epithelial-mesenchymal transition of alveolar epithelial cells - potential role in lung fibrosis. Thromb Haemost.

[17] Roche N, Stirling RG, Lim S, Oliver BG, Oates T, Jazrawi E, Caramori G, Chung KF (2003). Effect of acute and chronic inflammatory stimuli on expression of protease-activated receptors 1 and 2 in alveolar macrophages. J Allergy Clin Immunol.

[18] Bringardner BD, Baran CP, Eubank TD, Marsh CB (2008). The role of inflammation in the pathogenesis of idiopathic pulmonary fibrosis. Antioxid Redox Signal.

[19] Ariel A, Timor O (2013). Hanging inthe balance: endogenous anti-inflammatory mechanisms in tissue repair and fibrosis. J Pathol.

[20] Wynn TA, Barron L (2010). Macrophages: master regulators of inflammation and fibrosis. Semin Liver Dis.

[21] Lekkerkerker AN, Aarbiou J, van Es T, Janssen RA (2012). Cellular players in lung fibrosis. Curr Pharm Des.

[22] Fell CD, Martinez FJ, Liu LX, Murray S, Han MK, Kazerooni EA, Gross BH, Myers J, Travis WD, Colby TV, Toews GB, Flaherty KR (2010). Clinical predictors of a diagnosis of idiopathic pulmonary fibrosis. Am J Respir Crit Care Med.

[23] Raghu G, Weycker D, Edelsberg J, Bradford WZ, Oster G (2006). Incidence and prevalence of idiopathic pulmonary fibrosis. Am J Respir Crit Care Med.

[24] American Thoracic Society (2000). Idiopathic pulmonary fibrosis: diagnosis and treatment. International consensus statement. American Thoracic Society (ATS), and the European Respiratory Society (ERS). Am J Respir Crit Care Med.

[25] Gadepalli R, Kotla S, Heckle MR, Verma SK, Singh NK, Rao GN (2013). Novel role for p21-activated kinase 2 in thrombin-induced monocyte migration. J Biol Chem.

[26] Jenkins RG, Su X, Su G, Scotton CJ, Camerer E, Laurent GJ, Davis GE, Chambers RC, Matthay MA, Sheppard D (2006). Ligation of protease-activated receptor 1 enhances alpha(v) beta6 integrin-dependent TGF-beta activation and promotes acute lung injury. J Clin Invest.

[27] Nishimura SL (2009). Integrin-mediated transforming growth factor-beta activation, a potential therapeutic target in fibrogenic disorders. Am J Pathol.

[28] Jenkins G (2008). The role of proteases in transforming growth factor-beta activation. Int J Biochem Cell Biol.

[29] Pejler G, Lunderius C, Tomasini-Johansson B (2000). Macrophages synthesize factor X and secrete factorX/Xa-containing prothrombinase activity into the surrounding medium. Thromb Haemost.

[30] Scotton CJ (2009). Increasedlocal expression of coagulation factor X contributes to the fibrotic response in human and murine lung injury. J Clin Invest.

[31] Cooper DM, Pechkovsky DV, Hackett TL, Knight DA, Granville DJ, Granzyme K (2011). activates protease-activated receptor-1. PLoS One.

[32] Duitman J, Ruela-de-Sousa RR, Shi K, de Boer OJ, Borensztajn KS, Florquin S, Peppelenbosch MP, Spek CA (2014). Protease activated receptor-1 deficiency diminishes bleomycin-induced skin fibrosis. Mol Med.

[33] Sonin DL, Wakatsuki T, Routhu KV, Harmann LM, Petersen M, Meyer J, Strande JL (2013). Protease-activated receptor 1 inhibition by SCH79797 attenuates left ventricular remodeling and profibrotic activities of cardiac fibroblasts. J Cardiovasc Pharmacol Ther.

[34] Queiroz KC, Shi K, Duitman J, Aberson HL, Wilmink JW, van Noesel CJ, Richel DJ, Spek CA (2014). Protease-activated receptor-1 drives pancreatic cancer progression and chemoresistance. Int J Cancer.

[35] Duitman J, Schouten M, Groot AP, Borensztajn KS, Daalhuisen JB, Florquin S, van der Poll T, Spek CA (2012). CCAAT/enhancer-binding protein δ facilitates bacterial dissemination during pneumococcal pneumonia in a platelet-activating factor receptor-dependent manner. Proc Natl Acad Sci U S A.

